# How perceived scarcity predicted cooperation during early pandemic lockdown

**DOI:** 10.3389/fpsyg.2022.951757

**Published:** 2022-10-10

**Authors:** Claudia Civai, Marta Caserotti, Elisa Carrus, Inge Huijsmans, Enrico Rubaltelli

**Affiliations:** ^1^Division of Psychology, School of Applied Sciences, London South Bank University, London, United Kingdom; ^2^Dipartimento di Psicologia dello Sviluppo e della Socializzazione, Facolta’ di Psicologia, Universita’ degli Studi di Padova, Padova, Italy; ^3^Centre for Cognitive Neuroimaging, Donders Institute, Radboud University, Nijmegen, Netherlands

**Keywords:** scarcity, cooperation, social norms, prosociality, pandemic lockdown

## Abstract

Both material resources (jobs, healthcare), and socio-psychological resources (social contact) decreased during the COVID-19 pandemic. We investigated whether individual differences in perceived material and socio-psychological scarcity experienced during the pandemic predicted preference for cooperation, measured using two Public Good Games (PGGs), where participants contributed money or time (i.e., hours indoors contributed to shorten the lockdown). Material scarcity had no relationship with cooperation. Increased perceived scarcity of socio-psychological wellbeing (e.g., connecting with family) predicted increased preference for cooperation, suggesting that missing social contact fosters prosociality, whilst perceived scarcity of freedom (e.g., limited movement) predicted decreased willingness to spend time indoors to shorten the lockdown. The importance of considering individual differences in scarcity perception to best promote norm compliance is discussed.

## Introduction

The COVID-19 pandemic, especially during its peak, has led to an increase in job losses and overall loss in work hours, with many people struggling to receive an income as a result; in the United Kingdom, for example, a 2021 estimate suggests that, at the beginning of the year, unemployment rose to 5% for the first time in 5 years (Office for National Statistics, February 2021). Also, the strict COVID-related lockdown measures imposed worldwide, which include physical distancing and restrictions on leaving one’s property, have led to (and exacerbated pre-existing) difficulties with access to food and healthcare. Moreover, these restrictions have not only limited or decreased access to essential material resources, but they have likely led to the perception of a type of scarcity that is of a social and psychological nature: people were forced indoors without the opportunity to see family and friends, and, more broadly, enjoy the freedom to dispose of one’s own time. Therefore, the COVID-19 pandemic has likely increased both material and socio-psychological scarcity, or, at the very least, the perception of it; this, sadly, has offered the opportunity to investigate the psychological correlates of sudden perceived scarcity of a broad range of resources in individuals who may not usually experience this condition.

It has been established that scarcity influences cognitive abilities (Shah et al., 2012). Specifically, research found that, regardless of the resource domain (e.g., money, time, etc.), scarcity fosters the formation of a scarcity mindset that influences our actions and decisions across different cognitive domains (Mani et al., 2013; Tomm and Zhao, 2016; Shah et al., 2019). For example, experiencing a condition of scarcity leads to an increased tendency to borrow any kind of resource and causes myopic and impulsive behavior (Shah et al., 2012), prioritizing short-term over long-term gains. The inherent problem is that, under a scarcity mindset, it is difficult to plan, and the potential disadvantages of immediate benefit are overlooked (Shah et al., 2012). Considering a more social dimension, previous findings have led to contrasting conclusions: some authors have suggested that resource scarcity increases generosity toward others (Piff et al., 2010; Kraus et al., 2012), whilst others claim that it promotes selfish, or even antisocial, behaviors (Holland et al., 2012; Petersen et al., 2014; Prediger et al., 2014). Other findings have suggested that being reminded of resource scarcity promotes a competitive orientation, and a scarcity mindset can lead to behaviors that are not uniquely individualistic: in fact, it was suggested that the competitive orientation to ensure one’s own well-being could manifest itself in both increased selfishness and increased generosity, depending on the benefits associated with the self (Roux et al., 2015).

Research on the effects of scarcity usually involves comparing people with varying levels of access to resources, either as observed in the real world (rich vs. poor) (e.g., Mani et al., 2013; Prediger et al., 2014), or by experimentally manipulating people’s availability of resources or their perception of it (e.g., Shah et al., 2012; Durante et al., 2015; Roux et al., 2015; Huijsmans et al., 2019; see Cannon et al., 2019 for a review). Both approaches present their own issues: real-world investigations do not allow researchers to disentangle the effect of scarcity from other variables often associated with scarcity and poverty, such as chronic stress and mental ill-health (Lupien et al., 2001; Anand and Lea, 2011); laboratory manipulations, albeit allowing for more precise controls of confounds, may not accurately represent real-life consequences. Considering that the situation created by the COVID-19 pandemic may have caused people who do not usually experience scarcity to suddenly feel affected by it, the current study aims to investigate perceived scarcity in the real-world context.

This study examines the effect of two forms of perceived scarcity, material and socio-psychological, on prosocial behavior, specifically cooperation. Traditional economic theory is based on two main assumptions, which are that humans behave rationally, and follow their own self-interest, leaving little space for concepts such as prosociality in economic exchange. Nevertheless, decades of research in behavioral economics have shown that people do, in fact, take into consideration others’ states, wishes, and beliefs when making economic decisions (other-regarding preferences; Camerer, 1999): people engage in costly altruistic behaviors, such as third-party punishment (see, for example, Fehr and Fischbacher, 2004 or Buckholtz et al., 2008), and even reject lucrative deals when they are deemed to be unfair (as documented by the behavior in the Ultimatum Game, Güth et al., 1982; see Vavra et al., 2017 for a review on unfairness perception), showing that they are neither exclusively driven by self-interest, nor economically rational. Cooperation is a type of prosocial behavior that can sometimes be driven by self-interest: in fact, cooperation in repeated settings, i.e., when interactions occur repeatedly within the same group of people, can be explained by reciprocity, so that good behavior is reciprocated with good behavior (reciprocal altruism; Trivers, 1971). However, people often cooperate even when direct reciprocity is not possible, for example in one-shot situations, when repeated encounters are excluded and anonymity is guaranteed: these findings suggest that cooperation is not necessarily driven by reciprocity, and may be the result of specific personality traits, in that some people are instinctively more cooperative than others (for a review, see Van Dijk, 2015). In fact, it has been suggested that prosocial behavior, including cooperation, can be the intuitive response in social dilemmas (e.g., Rand et al., 2012; see Capraro, 2019 for a review of the debate), although recent findings suggest that both prosociality and self-interest can serve as intuitions in guiding behavior (Bago et al., 2021). In this historic moment, it seems to be particularly crucial to cooperate and prioritize the wellbeing of the group over the fulfillment of our immediate needs, as is understanding the factors that can contribute to increasing this behavior; if scarcity is associated with our ability to cooperate, then this needs to be considered when policies are communicated to people, especially those that rely on social norms and require the adherence of most people to be effective (e.g., lockdown rules).

To measure cooperation, we employed a Public Goods Game (PGG - Fehr and Gachter, 2000), where the resources contributed to a communal pot indicates the preference for cooperation. The PGG is not the only instrument that can be employed to study cooperative behaviors. Game Theory has developed different mathematical objects that have been translated into behavioral tasks, some of which can measure cooperation (see Civai and Hawes, 2016, for a review of games used in Neuroeconomics); probably the best known is the Prisoner’s Dilemma, a game where two players must simultaneously decide whether to cooperate with their partner, and lie to the police, or defect, and confess the crime. The payoff matrix of the game suggest that the optimal solution would be to defect; however, decades of experiments showed that many people decide to cooperate, even when the game is one-shot (e.g., Capraro et al., 2014). Another game used to investigate cooperation and trust is the Stag Hunt Game, where two players must decide between a small personal gain (hunt a hare) or a larger gain (hunt a stag), achievable only through cooperation. Deciding to cooperate and play Stag, as for the Prisoner’s Dilemma, includes an element of social risk that often people are willing to take (e.g., Belloc et al., 2019). In this study, the PGG was chosen for its structure that allows to include a large number of players, and for referencing communal resources, which makes it particularly suitable to be adapted to the real-life scenarios that we devised. Moreover, since we were interested in people’s intuitive response, rather than in a reciprocity-based cooperation, we employed a one-shot version of the PGG. Two scenarios were administered to tap separately into material and socio-psychological resources. Each scenario was contextualized to the COVID-19 lockdown and involved monetary bonuses for material resources (PGG-money) and socio-psychological bonuses in terms of “hours outdoors” for socio-psychological resources (PGG-time). The perception of scarcity was measured with two scales contextualized to the current pandemic: one tapping into material scarcity (e.g., receiving an income or getting groceries), and the other into socio-psychological scarcity (e.g., connecting with friends and family or managing time). It is important to note that these were not standard versions of the PGG. First of all, the scenarios were hypothetical, and therefore participants did not receive real incentives. Second, in order to prioritise relevance to the situation and ease of understanding, the structure of the PGG-time slightly deviated from the traditional PGG structure, which is instead the one adopted for the PGG-money, as detailed in the methods section. Since the aim of the study was to focus on the relationship between perceived scarcity and cooperation from a psychological perspective, we believe that the adopted games are suitable to measure our construct of interest, i.e., the willingness to cooperate, and therefore to answer our research question, despite deviating from the standard structure.

Given the known relationship between scarcity and negative affect (see Haushofer and Fehr, 2014; Cannon et al., 2019 for a review), Trait Emotional Intelligence (TEI; Petrides and Furnham, 2006) was considered as a potential moderator of the relationship between scarcity and cooperation. Indeed, TEI is found to regulate emotional experiences and can therefore be a reliable indicator of people’s ability to manage negative emotions and stress in the context of decision-making (Sevdalis et al., 2007; Agnoli et al., 2015). Furthermore, it was found that people with high TEI were more likely to use adaptive rather than maladaptive emotion regulation strategies (Mikolajczak et al., 2008), suggesting that high TEI increases the chance to adopt a coping strategy to handle a negative situation, without catastrophizing for every possible problem. Two further measures were added in order to isolate the predictive effects of scarcity from potentially confounding variables: the Communal Orientation Scale (COS) (Clark et al., 1987), which aims to capture individual differences in prosocial preferences, and the MacArthur Scale of Subjective Social Status (Adler and Stewart, 2007), given evidence suggesting that perceived high socio-economic status (SES) influences decision-making (e.g., Callan et al., 2011) and increases generosity (Smeets et al., 2015).

It was hypothesized that, (i) after controlling for communal orientation and subjective social status, perceived scarcity of resources would predict a decrease in cooperation (the amount contributed to the communal pot). Additionally, since perceived scarcity of resources fosters a “scarcity mindset,” it was hypothesized that (ii) the perception of scarcity in one domain (e.g., socio-psychological) may predict cooperation in the other domain (e.g., material, measure with PGG-money). It was also predicted that (iii) TEI would moderate the effect of perceived scarcity on cooperation, namely that an increased ability to control one’s own emotions (higher TEI) would lead to increased cooperation, even for higher perceived scarcity. Finally, it was hypothesized that (iv) the perceived change in resources between the present and the pre-pandemic condition would predict cooperation above and beyond perceived scarcity itself.

Data were collected at two different times: the first planned data collection (sample S1) was conducted at the beginning of May 2020, when the United Kingdom was still in its first strict lockdown (Great Britain Cabinet office 2020, March 23, 2020, withdrawn May 11, 2020), whilst the second (S2), which was not preregistered, was conducted at the end of August 2020. We believe that this deviation from the preregistration protocol is justified by the opportunity to explore whether cooperation would change with the evolving situation, characterized, in S2, by a potential increase in perceived material scarcity, elicited by the imminent ending of financial aids (Association of Taxation Technicians (ATT), 2020), and a potentially decreased socio-psychological scarcity, driven by the ease of the lockdown measures.

A between-participant factor was included for S1: for half of the sample, a cooperation nudge was used, where participants were explicitly told in the instructions that the PGG was a task measuring their prosocial preferences. Simple moral nudges, consisting in reminding participants about norms such as cooperation, have been shown to be effective in shifting behaviors in simple games (Capraro et al., 2019), therefore it was hypothesized that (v) the nudged group would cooperate more. However, no significant effect was found for the nudge manipulation, and therefore it was not applied to S2.

Hypotheses and methods were preregistered, and can be found here https://osf.io/xrm6p?view_only=7cf8d67b9b18464b831c6b145609a1c7.

## Materials and methods

### Participants

Participants were recruited through Prolific (www.prolific.co) and were paid £2.50. S1 was collected May 6^th^ - 8^th^, 2020; S2 August 27^th^-29^th^, 2020. Participants in S1 were excluded from S2. The demographic distribution was representative of the United Kingdom population in terms of age, sex and ethnicity based on 2011 United Kingdom Census, and this required a sample of at least 300 participants. S1 included 600 participants (300 for each nudge condition), whilst S2 included 300. Overall, 900 participants were considered, which, according to G*Power (Faul et al., 2007), was enough to detect a small effect size (*R*^2^ = 0.02) with 13 predictors, *α* = 0.05 and a power of 0.80. The mean age was 45.49 years (*SD* = 15.67 years), with 458 participants identifying as females, one as transgender, one as agender and one preferred not to say. Only data from participants who gave informed consent and completed the study within the suggested timeframe (20 min, +/− 2 SD) were kept; otherwise, participants were rejected, and new participants were recruited, as per Prolific guidelines.

The study was approved by the Ethics Panel of the School of Applied Sciences at London South Bank University, with protocol number ETH1920-0152.

### Materials

#### Public good game scenarios

The survey was conducted on Qualtrics. First, two scenarios of the PGG were administered, counterbalanced (full instructions are available here).^1^

##### PGG-money scenario

Participants were asked to imagine receiving a COVID-related financial bonus of £100 by the local authority. They were then asked to indicate how much of this money they would contribute back to the council, knowing that the council authority would double the donations and redistribute the resulting amount equally amongst the residents. In the instructions, participants were asked to imagine that the council had 50 residents, which was a number arbitrarily established for the sake of calculation. Participants were asked to indicate how much they wanted to contribute on a scale from £0 to £100, using a slider with increments of 10 and with 0 displayed as the starting point.

##### PGG-time scenario

Participants were asked to imagine receiving a bonus of 10 slots of 8 h each (*ca.* 10 days) from their local council, to be spent on outdoors activities, such as picnics in the park or trips to the beach, keeping social distancing measures. Participants were then asked how many of their 10 8-h slots they would contribute back to the council, knowing that for each slot spent at home, the lockdown restrictions would be lifted 16 h earlier for everyone. This was done to match the format of the PGG-money scenario, where the local council doubles the contribution to the communal pot of resources. In the instructions, it was explained that, in the event of no contributions being received (0 slots of 8 h) from the 50 residents, the lockdown measures would have been lifted after 365 days. On the other hand, if all 50 residents contributed everything (10 slots of 8 h, or 80 h), the lockdown would have been lifted 334 days earlier, or in 31 days. If 49 residents contributed everything and one resident contributed nothing, the lockdown would have been lifted after 38 days for everyone, but the one resident who contributed nothing would have benefitted from an additional 10 days (80 h) outside. The threshold of 365 days was chosen because we believed it would have been easy to picture for participants, it being one year. Participants were asked to indicate how many 8-h slots they wanted to contribute on a scale from 1 to 10, using a slider with increments of 1 and with 0 displayed as the starting point.

In S1, we introduced a nudge manipulation before the presentation of the PGG: half the sample was informed that the questions aimed to measure how much participants valued cooperation to achieve maximum benefit for everyone (nudge). The other half were informed that the purpose of the questions was to assess their preference for how to dispose of certain resources (no nudge). In S2, the nudge condition was removed, and only the no nudge instructions were presented.

As mentioned in the introduction, there are some differences between the adopted PGG and the standard PGG. First of all, both scenarios were hypothetical, i.e., not incentivized: given that only one of the two scenarios involved monetary considerations and preference for cooperation in economic terms, we preferred to eliminate the potential confound of an economic reward from both scenarios. Moreover, a review of the literature showed that behavior in hypothetical versus incentivized PGG scenarios does not differ substantially (Gillis and Hettler, 2007). Second, the structures of the PGG-time and PGG-money differed slightly: in fact, for the PGG-time, the communal pot was not divided in equal parts among participants; instead, all participants would benefit from the total amount of the communal pot. We believed that this would have been easier to understand, and would still have measured our construct of interest, i.e., the willingness to cooperate; in fact, as it happens for the standard PGG (and for the PGG-money), if one contributes zero, they will still benefit from the communal resources, whilst if one contributes something, they increase the chance of shortening the lockdown for everyone, including themselves.

#### Scarcity measures

Two scarcity dimensions were measured by asking participants to indicate how much they agreed with the statement “I have (or foresee) problems” on a 5-point Likert scale (1 = Strongly disagree to 5 = Strongly agree), in response to a few items. Each of the two dimensions contained five items, where higher scores indicated higher perceived scarcity.

The material scarcity (MatScar) dimension included items related to financial resources as well as material daily life resources, specifically groceries and access to healthcare, which are salient in the pandemic context. The five items were: “Receiving my usual income/allowance” (MatScar_1); “Getting grocery items” (MatScar_2); “Paying bills” (MatScar_3); “Accessing healthcare” (MatScar_4); “Keeping my job” (MatScar_5).

The socio-psychological scarcity (SocScar) dimension included items that encompass resources that are clearly social (family and friends) as well as others that are more psychological and related to movement restrictions (coping with restrictions or managing time). The items were: “Connecting with friends and family” (SocScar_1); “Spending time doing the things I enjoy the most” (SocScar_2); “Looking after my physical wellbeing” (SocScar_3); “Coping with current restrictions of movement” (SocScar_4); “Managing time” (SocScar_5).

Two additional questions asked participants to indicate whether, compared to the time before the COVID-19 pandemic, they thought their resources (material or socio-psychological) had decreased, on a scale from 1 (substantially decreased) to 5 (substantially increased). Since higher scores indicated increased resources, the scores were reversed to keep the interpretation consistent with the other two scarcity dimensions.

#### Additional questionnaires

Three additional questionnaires were administered. The Trait Emotional Intelligence Questionnaire short form (TEIQue – SF; Petrides and Furnham, 2006) is a 30-item questionnaire (e.g., “I often find it difficult to adjust my life according to the circumstances”), to which participants agree on a 7-point Likert scale, and where higher scores indicate higher trait emotional intelligence. The Communal Orientation Scale (COS; Clark et al., 1987) is a 14-item scale (e.g., “When making a decision, I take other people’s needs and feelings into account”), to which participants respond on a 7-point Liker scale, and where higher scores indicate a higher communal orientation. The MacArthur Scale of Subjective Social Status (Adler and Stewart, 2007) is a visual scale where participants indicate the rung on which they see themselves standing on the social ladder, where higher scores indicate higher subjective social status.

### Design

A correlational design was used to investigate whether perceived material and socio-psychological scarcity, and their interaction with TEIQue, predicted cooperation on material (PGG-money) and socio-psychological (PGG-time) resources. Prosocial orientation (COS) and subjective social status (MacArthur) were added as covariates. Sample was added as an additional binary predictor to consider the effects of the time of data collection on cooperation (May or August). For S1, Nudge was added as a binary predictor.

### Procedure

Participants were recruited through Prolific and re-directed to Qualtrics to complete the survey, after giving informed consent. The PGG scenarios were presented first, in a counterbalanced order. Then, the scarcity scales were presented, followed by the TEIQue, COS and MacArthur, also counterbalanced. At the end, demographics were collected: participants were asked their age, gender, nationality, income, education, and working status, as well as pandemic-specific questions such as their living condition (living alone or with others), whether they were a key worker (e.g., nurse, grocery store salesperson, etc), had any vulnerability (e.g., pre-existing conditions such as asthma or diabetes), and/or had been affected by COVID-19 symptoms. Finally, participants read a debrief sheet and were re-directed to Prolific for payment. Attentional checks were included only for S2, where participants were asked to report which version of the PGG they had done last (money or time): only 11 people out of 300 failed the test but their data were not removed because excluding them from the sample did not change the results; most importantly, it ensured consistency with S1 and the preregistration.

## Results

The dataset and the analysis scripts for this study can be found in the Open Science Framework repository.^2^

### Exploratory factor analysis of scarcity scales

**Figure 1:**
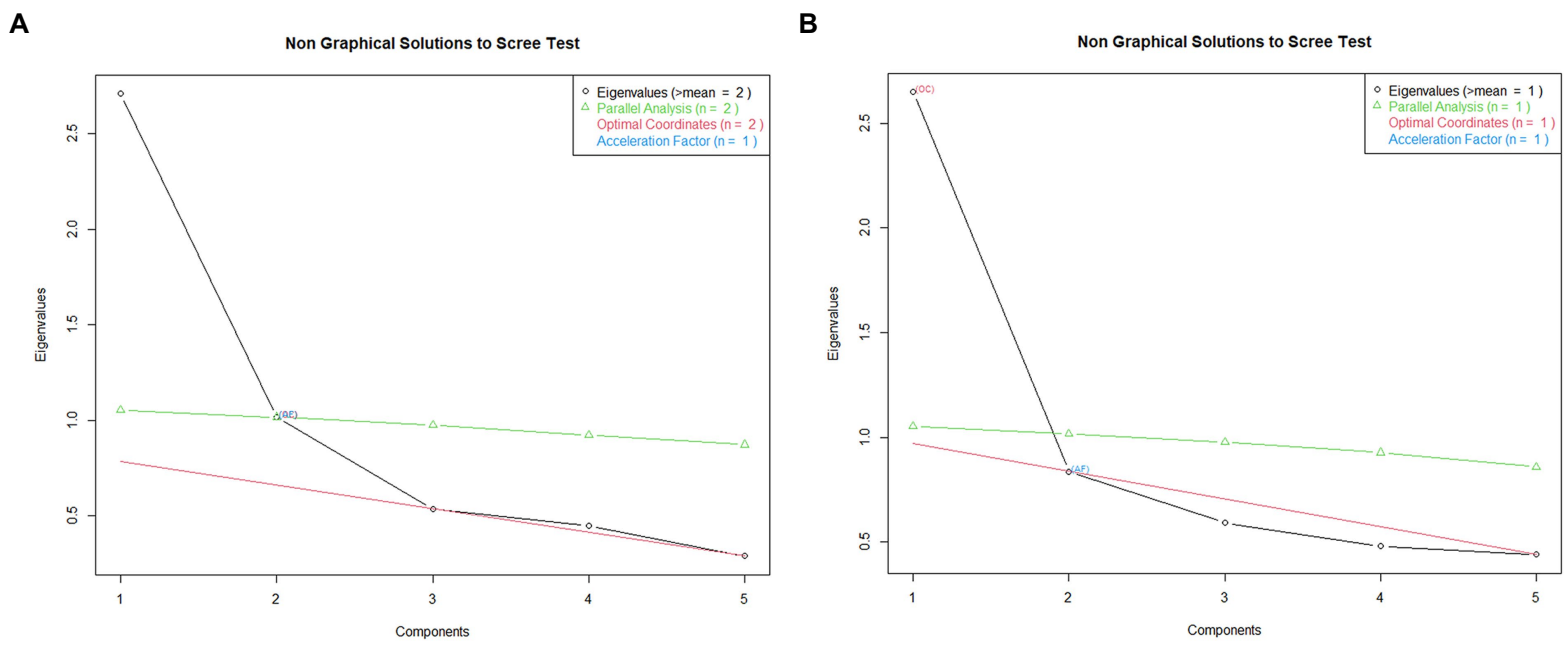
Scree plot of the EFA: **(A)** Material Scarcity factors; **(B)** Socio-Psychological Scarcity factors.

**Figure 2:**
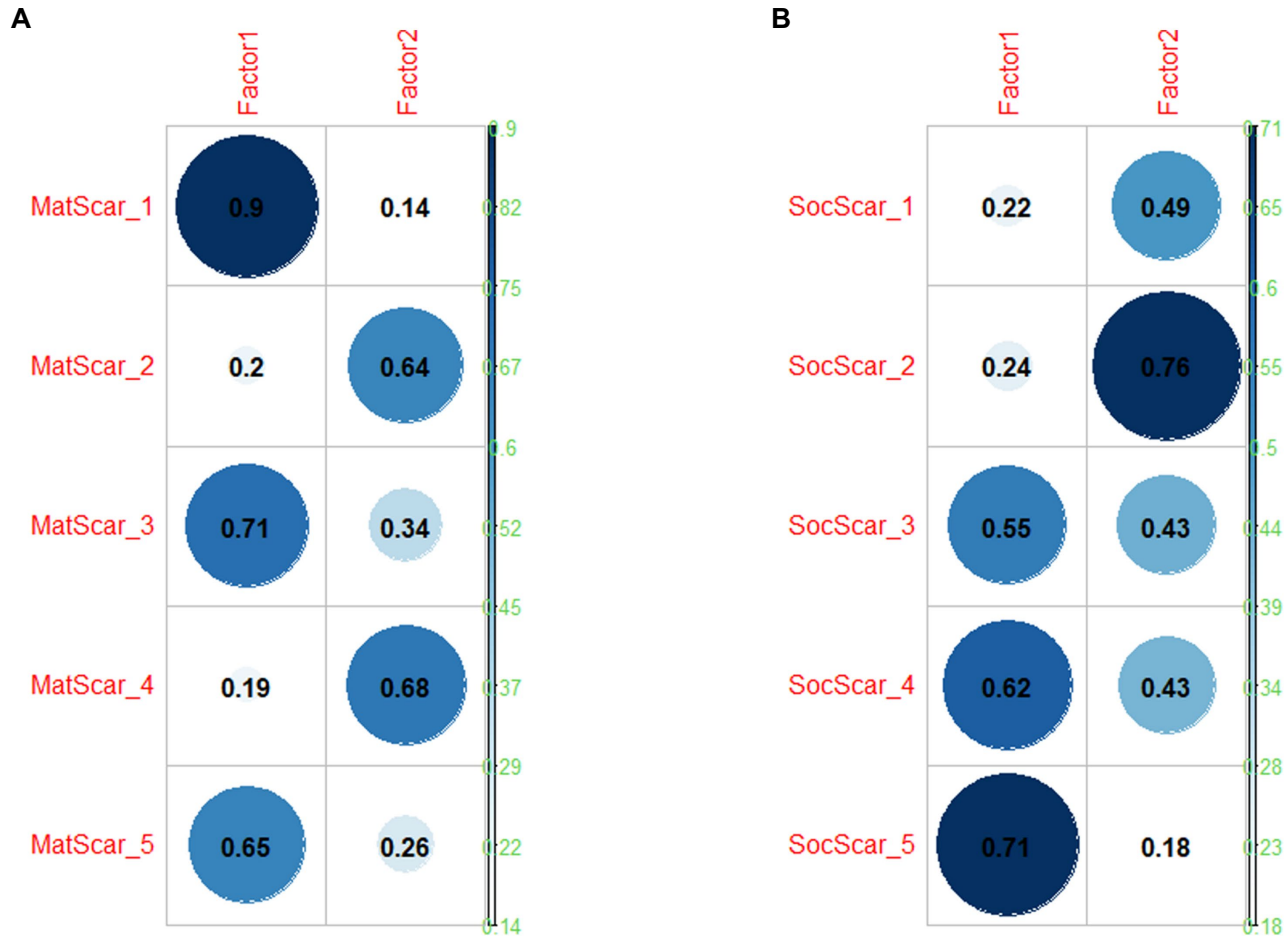
Factor loadings for each item: **(A)** Material Scarcity loadings; **(B)** Socio-Psychological Scarcity loadings.

Two exploratory factor analyses (EFA) were performed (*factanal* R function, *psych* package, Revelle, 2020; maximum likelihood estimation method, “varimax” rotation) on the two scarcity dimensions. For each dimension, a two-factor solution was selected, based on three considerations: it captured the theoretical differences among the items administered, as explained in the Materials section; a visual inspection of the scree plots (*nfactor* package in R, Raiche and Magis, 2020) showed that the plot levelled off after the second factor for both dimensions (Figures 1A,B), although this is clearer for Material than for Socio-psychological Scarcity; the two-factor models fitted the data better than the one-factor. In fact, for both dimensions, the hypothesis testing suggested not to reject the null hypotheses that the 2-factor model fits the data (material: Chi-square = 2.88, *p* = 0.08; socio-psychological: Chi-square = 0.47, *p* = 0.5). The final cumulative variance was 0.58 (Factor 1 = 0.36; Factor 2 = 0.22) for material scarcity and 0.50 (Factor 1 = 0.26; Factor 2 = 0.24) for socio-psychological scarcity (as a rule of thumb, “for the number of ‘real’ factors and components, the proportion [of variance accounted for] should be at least 0.50.” (Merenda, 1997, p. 158)). The loadings for each item on each factor are represented in Figure 2A (material) and Figure 2B (socio-psychological).

For material scarcity, the factors are theoretically compatible with the distinction between scarcity of financial resources, i.e., job, income and means to paying bills (MatScar_1, MatScar_3, and MatScar_5: Finance) and scarcity of daily life items, i.e., groceries or accessing health care (MatScar_2 and MatScar_4: Daily Life). For socio-psychological scarcity, the two factors were related to scarcity of freedom, i.e., coping with restrictions of movement, looking after physical wellbeing, and time management (SocScar_3, SocScar_4, and SocScar_5: Freedom) and scarcity of socio-psychological wellbeing, i.e., connecting with friends and family and enjoying life (SocScar_1 and SocScar_2: Socio-Psychological Wellbeing).

Cronbach’s alpha for Finance scarcity (3 items) was 0.82 (high; Gliem and Gliem, 2003), whilst for Daily Life scarcity (2 items) was 0.64 (moderate); the alpha for Freedom scarcity (3 items) was 0.74 (adequate), whilst for Socio-Psychological Wellbeing scarcity (2 items) was.59 (moderate/poor). Composite scores were then created for each of the factors, based on the average of the items which had their primary loadings on each factor.

### Descriptive statistics and comparison across samples

On average, participants experienced some scarcity (Table 1). A comparison between S1 and S2 showed that the Daily Life scarcity was the only measure that significantly differed across samples after Bonferroni’s correction, being higher in S1 (t (590) = 4.07, *p* < 0.001); (Table 2).

**Table 1:** Descriptive statistics of the key variables.

**Variables**	***M***	***SD***	**Median**	**Sample range**
Age	45.49	15.67	45.50	18–87
PGG_money	64.30	36.43	70.00	0–100
PGG_time	7.89	2.90	9.00	0–10
COS	51.25	8.22	52.00	16–70
MacArthur	5.42	1.71	6.00	1–10
TEIQue	142.92	25.75	144.00	62–205
DailyLife	2.83	1.09	3.00	1–5
Finance	2.45	1.20	2.33	1–5
Wellbeing	3.22	1.11	3.50	1–5
Freedom	2.71	1.00	2.67	1–5
Mat_change	3.30	0.77	3.00	1–5
Soc_change	3.38	1.02	3.00	1–5

*N* = 900.

**Table 2:** Comparison between perceived scarcity measures in S1 (*N* = 600) and S2 (*N* = 300).

**Scarcity variables**	**MS1**	**MS2**	**SD S1**	**SD S1**	***t*-value**	**95% CI**	**Value of *p***
DailyLife	2.93	2.62	1.07	1.09	4.07	[0.16, 0.46]	<0.001*
Finance	2.39	2.58	1.19	1.2	−2.29	[−0.36, −0.03]	0.023
Wellbeing	3.27	3.11	1.12	1.07	2.07	[0.01, 0.31]	0.039
Freedom	2.7	2.73	1.03	0.94	−0.47	[−0.17, 0.1]	0.639
Mat_change	3.34	3.23	0.76	0.79	2	[0.01, 0.22]	0.046
Soc_change	3.42	3.29	1	1.06	1.83	[−0.01, 0.28]	0.067

* Bonferroni corrected value of *p* threshold 0.008.

### Predictive models of cooperation

Two hierarchical linear regressions were run for each outcome variable (*reghelper* R package, Hughes, 2020; confidence intervals were calculated with *sjplot,* Lüdecke, 2021); given the large sample size (*N* = 900) and considering the robustness of the linear models against violations of normality (Knief and Forstmeier, 2021), linear regression was used. Model 1 included the covariates and the time of data collection (S1 or S2); model 2 included the four material and socio-psychological scarcity subscales; model 3 included the interaction term between the scarcity subscales and TEIQue; and model 4 included the two questions related to the change in perceived scarcity, material (mat_change) and socio-psychological (soc_change). For S1, Nudge was included as a between-participant factor, and therefore model 5 was run only on S1 data (Tables 3, 4). All variables were scaled to obtain standardized beta coefficients. The intercorrelations among items are presented in Table 5.

**Table 3:** (a) Hierarchical regression for the outcome variable PGG-money: standardized *β* coefficients, 95% CIs and change statistics for four models (*N* = 900).

**Mod**	**Predictor**	***β***	**95% CI**	**Value of *p***	***R*** ^**2**^	***F***	***p***	***R*** ^**2**^_***c***_	***F***_***c***_	***p***_***c***_
1					0.11	37.47	<0.001			
	Intercept	0.08	[0.01, 0.16]	0.034^*^						
	MacArthur	0.13	[0.07, 0.20]	<0.001^***^						
	COS	0.28	[0.22, 0.35]	<0.001^***^						
	Sample_1	−0.25	[−0.38, −0.11]	<0.001^***^						
2					0.12	17.15	<0.001	0.01	1.82	0.124
	Intercept	0.08	[0.01, 0.16]	0.034^*^						
	MacArthur	0.12	[0.07, 0.20]	<0.001^***^						
	COS	0.28	[0.22, 0.35]	<0.001^***^						
	Sample_1	−0.25	[−0.38, −0.11]	<0.001^***^						
	DailyLife	−0.06	[−0.13, 0.01]	0.095						
	Finance	0.02	[−0.05, 0.09]	0.602						
	Wellbeing	0.08	[0.01, 0.16]	0.025*						
	Freedom	−0.03	[−0.11, 0.04]	0.375						
3					0.13	10.57	<0.001	0.01	1.32	0.255
	Intercept	0.09	[0.01, 0.16]	0.022*						
	MacArthur	0.12	[0.07, 0.20]	<0.001^***^						
	COS	0.28	[0.22, 0.35]	<0.001^***^						
	Sample_1	−0.25	[−0.38, −0.11]	<0.001^***^						
	DailyLife	−0.06	[−0.13, 0.01]	0.133						
	Finance	0.01	[−0.05, 0.09]	0.748						
	Wellbeing	0.08	[0.01, 0.16]	0.032*						
	Freedom	−0.03	[−0.11, 0.04]	0.397						
	TEIQ	0.01	[−0.06, 0.08]	0.757						
	TEIQ*DailyLife	−0.06	[−0.13, 0.08]	0.072						
	TEIQ*Finance	0.06	[−0.01, 0.13]	0.118						
	TEIQ*Wellbeing	0.00	[−0.07, 0.08]	0.931						
	TEIQ*Freedom	0.05	[−0.02, 0.12]	0.176						
4					0.13	9.22	<0.001	0.00	1.07	0.343
	Intercept	0.09	[0.01, 0.16]	0.020^*^						
	MacArthur	0.11	[0.07, 0.20]	0.001^**^						
	COS	0.28	[0.22, 0.35]	<0.001^***^						
	Sample_1	−0.25	[−0.38, −0.11]	<0.001^***^						
	DailyLife	−0.05	[−0.13, 0.01]	0.147						
	Finance	0.01	[−0.05, 0.09]	0.747						
	Wellbeing	0.09	[0.01, 0.16]	0.023^*^						
	Freedom	−0.02	[−0.11, 0.04]	0.597						
	TEIQ	0.01	[−0.06, 0.08]	0.810						
	TEIQ*DailyLife	−0.07	[−0.13, 0.08]	0.058						
	TEIQ*Finance	0.06	[−0.01, 0.13]	0.109						
	TEIQ*Wellbeing	0.00	[−0.07, 0.08]	0.967						
	TEIQ*Freedom	0.05	[−0.02, 0.12]	0.172						
	Mat_change	−0.01	[−0.08, 0.05]	0.711						
	Soc_change	−0.05	[−0.12, 0.02]	0.173						
**(b) Regression for the outcome variable PGG-money including Nudge predictor: standardized *β* coefficients, 95% Cis, only S1 (*N* = 600).**										
	**Predictor**		**95% CI**	**Value of *p***	***R*** ^**2**^	***F***	***p***			
	Intercept		[0.03, 0.19]	0.293	0.15	7.42	<0.001			
	MacArthur		[0.05, 0.22]	<0.001^***^						
	COS		[0.25, 0.41]	<0.001^***^						
	DailyLife		[−0.14, 0.03]	0.171						
	Finance		[−0.05, 0.13]	0.321						
	Wellbeing		[−0.02, 0.16]	0.132						
	Freedom		[−0.11, 0.08]	0.733						
	TEIQ		[−0.11, 0.07]	0.864						
	TEIQ*DailyLife		[−0.15, 0.02]	0.133						
	TEIQ*Finance		[−0.04, 0.13]	0.154						
	TEIQ*Wellbeing		[−0.11, 0.06]	0.513						
	TEIQ*Freedom		[−0.05, 0.14]	0.098						
	Mat_change		[−0.09, 0.08]	0.939						
	Soc_change		[−0.11, 0.06]	0.673						
	Nudge		[−0.06, 0.23]	0.242						

Outcome variable: PGG-money, *p < 0.05; **p < 0.01; ***p < 0.001.

**Table 4:** (a) Hierarchical regression for the outcome variable PGG-time: standardized *β* coefficients, 95% CIs and change statistics for four models (*N* = 900).

**Mod**	**Predictor**	***β***	**95% CI**	**Value of *p***	***R*** ^**2**^	***F***	***p***	***R*** ^**2**^_**c**_	***F***_***c***_	***p***_***c***_
1					0.01	3.58	0.014			
	Intercept	0.02	[−0.06, 0.10]	0.703						
	MacArthur	0.02	[−0.04, 0.09]	0.455						
	COS	0.10	[0.04, 0.17]	0.001**						
	Sample_1	−0.05	[−0.19, 0.09]	0.509						
2					0.02	3.01	0.004	0.01	2.54	0.038*
	Intercept	0.01	[−0.06, 0.10]	0.748						
	MacArthur	0.00	[−0.04, 0.09]	0.920						
	COS	0.10	[0.04, 0.17]	0.002**						
	Sample_1	−0.04	[−0.19, 0.09]	0.585						
	DailyLife	−0.04	[−0.12, 0.04]	0.299						
	Finance	0.01	[−0.07, 0.08]	0.878						
	Wellbeing	0.10	[0.03, 0.18]	0.009**						
	Freedom	−0.10	[−0.18, −0.02]	0.015*						
3					0.02	1.80	0.044	0.00	0.14	0.984
	Intercept	0.02	[−0.06, 0.10]	0.663						
	MacArthur	0.00	[−0.04, 0.09]	0.922						
	COS	0.10	[0.04, 0.17]	0.003**						
	Sample_1	−0.04	[−0.19, 0.09]	0.594						
	DailyLife	−0.04	[−0.12, 0.04]	0.313						
	Finance	0.00	[−0.07, 0.08]	0.926						
	Wellbeing	0.10	[0.03, 0.18]	0.011*						
	Freedom	−0.10	[−0.18, −0.02]	0.015*						
	TEIQ	−0.01	[−0.08, 0.07]	0.891						
	TEIQ*DailyLife	−0.01	[−0.08, 0.06]	0.733						
	TEIQ*Finance	0.02	[−0.05, 0.09]	0.599						
	TEIQ*Wellbeing	0.00	[−0.08, 0.07]	0.958						
	TEIQ*Freedom	0.02	[−0.06, 0.10]	0.634						
4					0.03	1.68	0.055	0.00	0.95	0.386
	Intercept	0.02	[−0.06, 0.10]	0.641						
	MacArthur	0.00	[−0.04, 0.09]	0.999						
	COS	0.10	[0.04, 0.17]	0.003*						
	Sample_1	−0.04	[−0.19, 0.09]	0.537						
	DailyLife	−0.04	[−0.12, 0.04]	0.338						
	Finance	0.00	[−0.07, 0.08]	0.927						
	Wellbeing	0.11	[0.03, 0.18]	0.008**						
	Freedom	−0.09	[−0.18, −0.02]	0.038*						
	TEIQ	−0.01	[−0.08, 0.07]	0.841						
	TEIQ*DailyLife	−0.02	[−0.08, 0.06]	0.661						
	TEIQ*Finance	0.02	[−0.05, 0.09]	0.573						
	TEIQ*Wellbeing	0.00	[−0.08, 0.07]	0.923						
	TEIQ*Freedom	0.02	[−0.06, 0.10]	0.625						
	Mat_change	−0.01	[−0.08, 0.06]	0.759						
	Soc_change	−0.05	[−0.06, 0.10]	0.193						
**(b) Regression for the outcome variable PGG-time including Nudge predictor: standardized *β* coefficients, 95% Cis, only S1 (*N* = 600).**										
	**Predictor**		**95% CI**	**Value of *p***	***R* ^2^**	***F***	***p***			
	Intercept		[−0.09, 0.14]	0.716	0.04	1.68	0.05			
	MacArthur		[−0.05, 0.13]	0.360						
	COS		[0.05, 0.22]	0.002**						
	DailyLife		[−0.13, 0.05]	0.377						
	Finance		[−0.11, 0.08]	0.770						
	Wellbeing		[0.03, 0.22]	0.011*						
	Freedom		[−0.18, 0.02]	0.125						
	TEIQ		[−0.14, 0.12]	0.570						
	TEIQ*DailyLife		[−0.10, 0.08]	0.728						
	TEIQ*Finance		[−0.10, 0.09]	0.960						
	TEIQ*Wellbeing		[−0.11, 0.08]	0.628						
	TEIQ*Freedom		[−0.04, 0.16]	0.329						
	Mat_change		[−0.05, 0.14]	0.450						
	Soc_change		[−0.14, 0.05]	0.318						
	Nudge		[−0.16, 0.16]	0.998						

Outcome variable: PGG-time, *p < 0.05; **p < 0.01; ***p < 0.001.

**Table 5:** Intercorrelations among regression variables (*r* coefficients [lower and upper 95% CI]).

**Variables**	**PGG_money**	**PGG_time**	**COS**	**MacArthur**	**DailyLife**	**Finance**	**Wellbeing**	**Freedom**	**TEIQue**	**Mat_change**	**Soc_change**
PGG_money	1	0.253^**^ [0.19,0.31]	0.285^**^ [0.22,0.34]	0.145^**^ [0.08,0.21]	−0.059 [0.12,0.01]	−0.050 [−0.11,0.02]	0.076^*^ [0.01,0.14]	−0.020 [0.01,0.14]	0.138^**^ [0.07,0.20]	−0.032 [−0.10,0.03]	−0.056 [−0.12, 0.01]
PGG_time		1	0.104^**^ [0.04,0.17]	0.029 [−0.01, 0.09]	−0.046 [−0.11, 0.02]	−0.028 [−0.09, 0.04]	0.048 [−0.02, 0.11]	−0.056 [−0.12, 0.01]	0.047 [−0.02, 0.11]	−0.025 [−0.09, 0.04]	−0.057 [−0.12, 0.01]
COS			1	0.051 [−0.01, 0.12]	−0.018 [−0.08, 0.05]	−0.016 [−0.08, 0.02]	0.048 [−0.02, 0.11]	0.018 [−0.05, 0.08]	0.297^**^ [0.24,0.36]	0.006 [−0.06, 0.07]	−0.050 [−0.11, 0.02]
MacArthur				1	−0.203^**^ [−0.26, −0.14]	−0.251^**^ [−0.31, −0.25]	0.004 [−0.06, 0.07]	−0.144^**^ [−0.21, −0.08]	0.345^**^ [0.29,0.40]	−0.202^**^ [−0.26,−0.14]	−0.097^**^ [−0.16, −0.03]
DailyLife					1	0.417^**^ [0.36,0.47]	0.235^**^ [0.17,0.30]	0.324^**^ [0.26,0.38]	−0.233^**^ [−0.29, −0.17]	0.186^**^ [0.12, 0.25]	0.148^**^ [0.08,0.21]
Finance						1	0.115^**^ [0.05, 0.18]	0.250^**^ [0.19, 0.31]	−0.160^**^ [−0.22, −0.10]	0.367^**^ [0.31, 0.42]	0.036 [−0.03, 0.10]
Wellbeing							1	0.522^**^ [0.47, 0.57]	−0.178^**^ [−0.24, −0.18]	0.032 [−0.03, 0.10]	0.269^**^ [0.21, 0.33]
Freedom								1	−0.304^**^ [−0.36, −0.24]	0.111^**^ [0.05, 0.18]	0.336^**^ [0.28,0.39]
TEIQue									1	−0.120^**^ [−0.18,−0.05]	−0.172^**^ [−0.23, −0.11]
Mat_change										1	0.131^**^ [0.07, 0.20]
Soc_change											1

* *p* < 0.05;

** *p* < 0.01;

*** *p* < 0.001.

For PGG-money, the effect size of the full model is medium (*R*^2^ = 0.13). COS (est. = 1.23; beta = 0.28; *p* < 0.001) and MacArthur (est. = 2.51; beta = 0.11, *p* = 0.001) positively predicted the monetary contribution, while Sample negatively predicted the amount (est. = −9.37; beta = −0.25; *p* < 0.001), indicating that participants in S2 (August 2020) contributed less than those in S1 (May 2020). Socio-Psychological Wellbeing positively predicted the amount contributed (est. = 2.95; beta = 0.09; *p* = 0.02), although the *F*-change of the model (model 2) was not significant (Table 3).

For PGG-time, the effect size of the full model is small (*R*^2^ = 0.03). COS positively predicted contribution (est. = 0.03; beta = 0.10; *p* = 0.003). Perceived scarcity of Freedom (est. = −0.25; beta = −0.09; *p* = 0.04) and of Socio-psychological Wellbeing (est. = 0.28; beta = 0.11; *p* = 0.006) significantly predicted time contributed (*F*-change = 2.54, *p* = 0.038), but in opposite directions: higher scarcity of Freedom predicted fewer time slots contributed, whilst higher scarcity of Socio-psychological Wellbeing predicted more time slots contributed (Table 4).

For both the PGG-money and PGG-time, the median is very high (Table 2): 50% of the participants decided to contribute more than £70 in the PGG-money, and 50% of participants decided to contribute more than 9 time slots in the PGG-time. For this reason, we ran two exploratory logistic regression models, using the median as cut-off point. The results, reported in the Supplementary materials, confirmed the findings from the pre-registered linear models reported above. The Supplementary materials also report a logit model including only the demographic variables as predictors.

## Discussion

The aim of this study was to investigate whether perceived scarcity of resources could predict cooperative behavior. The resources involved in the cooperation tasks, as well as those considered for perceived scarcity, were contextualized to the COVID-19 pandemic, and encompassed material (money, job, and access to daily life items) and socio-psychological (time, freedom, and social connections) items. Two samples were collected, one in May 2020 and one in August 2020, to capture differences related to changes in lockdown rules and availability of government financial aids.

For each scarcity domain, two aspects were captured by the items administered. For the material domain, the items captured the perceived scarcity of financial resources, such as income and job security, and the perceived scarcity of daily life items, like groceries and healthcare. For context, the study was run in the United Kingdom, which adopts a universal healthcare system where access to healthcare is not necessarily linked to financial scarcity, as it might be the case in other countries, such as the United States, for example. For the socio-psychological domain, the items captured the lack of socio-psychological wellbeing, encompassing social connections and life enjoyment, and the lack of freedom to move and dispose of one’s own time (e.g., the demands of home-schooling while also working from home created a situation where parents struggled to manage time). On average, participants perceived a change in resource availability compared to the pre-pandemic situation, and experienced some scarcity, especially in relation to socio-psychological wellbeing. Perceived scarcity did not significantly change between the two samples, except for daily life resources, which were perceived as significantly scarcer during the first lockdown in May compared to August. On the other hand, financial resources were perceived as scarcer in August; this was hypothesized since financial aid programs were coming to an end in October, but this comparison did not survive the statistical correction for multiple comparisons. Nevertheless, this may partly explain why participants contributed significantly more money to the communal pot in May compared to August.

As far as the relationship between scarcity and cooperation is concerned, it was hypothesized that (i) perceived scarcity would negatively predict cooperation, above and beyond individual prosocial orientation and subjective social status, and that (ii) the scarcity mindset created by the lack of resources in one domain (e.g., socio psychological) would predict cooperation on resources from both domains (i.e., time and money). The current findings partially support these hypotheses. As expected, a higher communal orientation, indicating how much an individual believes in mutual help, and a higher subjective social status predicted higher financial cooperation, whilst only communal orientation positively predicted time cooperation. Firstly, this confirms that these public goods games scenarios captured prosocial behavior; moreover, it supports previous findings showing that prosociality predicts cooperative health behaviors during the current pandemic (Campos-Mercade et al., 2021; Jordan et al., 2021). This result also shows that people who perceive themselves higher up in the social ladder are more inclined to cooperate and engage in giving behavior: this is in line with findings showing that low SES is associated with decreased cooperative behavior in children (Sheehy-Skeffington and Rea, 2017), and high SES is associated with increased giving behavior (Smeets et al., 2015).

Whilst the predictive effect of material scarcity was small and non-significant, contrary to our expectations, perceived scarcity of socio-psychological wellbeing positively predicted both monetary and time cooperation, in that higher perceived scarcity predicted higher cooperation. A positive relationship between scarcity and self-reported (not actual) prosociality has been previously reported (Petersen et al., 2014). A potential explanation is proposed in Cannon et al. (2019), who suggest that people who perceive scarcity and a lack of control over their own resources may identify more strongly with the group to increase a global sense of control, leading to increased cooperation. An alternative explanation is that people who perceive socio-psychological scarcity and favor social activities, may be more prone to favor cooperation over self-interest for achieving the greater good. Here, the effect of socio-psychological wellbeing scarcity was significant even after controlling for communal orientation; however, this relationship may be explained by another underlying factor, such as the Enthusiasm facet of Extraversion, which has been associated with sociability and positive affect (DeYoung et al., 2007) and has been found to positively predict cooperation (Hirsh and Peterson, 2009). Perceived scarcity of freedom, which does not necessarily entail any social element, led to the opposite effect, supporting our original hypothesis: higher perceived scarcity predicted a lower amount of free time slots contributed to the communal pot to shorten the lockdown for everyone. This is in line with previous research showing that, similarly to low SES and low perceived power, the perception of higher scarcity of resources is associated with higher temporal discounting, with people being more inclined to choose an immediate smaller reward over a larger delayed one (Callan et al., 2011; Sheehy-Skeffington, 2020); here, higher temporal discounting could have led participants to opt for free riding rather than reflecting on the long-term benefits of cooperation, and therefore could have prevented them from being connected to the future self and from trusting future redistribution of the resources (Joshi and Fast, 2013). One reason why this effect was not observed for perceived scarcity of socio-psychological wellbeing may also be that, in the PGG-time, participants were explicitly told that they could spend their free time-slots outdoors but respecting social distancing measures: this implied limited social contacts and ruled out events involving crowds, and therefore may not have been a strong enough incentive for those experiencing socio-psychological wellbeing scarcity to forgo the common good for their own self-interest.

This study also looked at perceived scarcity before and during the pandemic, hypothesizing that (iv) a perceived change in scarcity compared to pre-pandemic conditions, rather than perceived scarcity *per se*, would be a stronger predictor of cooperation. The current findings fail to support this hypothesis: even if participants report a perceived change in the availability of resources, this does not predict cooperation over and above the other measures of scarcity. One explanation might be that only one question on scarcity change was administered, without distinguishing between financial vs. daily life scarcity (material domain) and between socio-psychological wellbeing vs. freedom (socio-psychological domain): considering that these subscales predict cooperation in opposite directions, the general question has probably failed to capture these different and opposite contributions.

Since scarcity has been associated with negative affect, and TEI moderates the effects of negative affect on decision-making, it was hypothesized that (iii) TEI would moderate the effect of perceived scarcity on cooperation. In this study, TEI negatively correlated with perceived scarcity, but no significant moderation effect of TEI on the relationship between scarcity and cooperation was found. It is worth noting that Agnoli et al. (2015) report an effect of TEI on decision-making only in affect-rich situations, such as when helping others: in their study, participants received feedback on whether they managed to save the lives of children in need. In the current study, PGG scenarios have probably failed to elicit a strong affective response, and therefore TEI was not involved in decision-making. It is also worth noting that perceived scarcity might not have been related to negative affect, since not all studies in the literature report this relationship (e.g., Roux et al., 2015).

Lastly, based on Capraro et al. (2019), we used a nudge manipulation in S1. However, against our prediction that (v) a simple nudge presented before the PGG would influence cooperation, no such effect was observed, and therefore the nudge manipulation was subsequently dropped in S2. There is a key difference between the current study and Capraro et al.: in the latter, participants were required to respond to the question “what do you personally think is the morally right thing to do in this situation?”; in our study, participants simply read a statement that described the subsequent tasks as games designed to measure their cooperative and prosocial preferences, which may not have been salient enough. Therefore, these nudges may require an active engagement from the participant to work and be more explicitly stressing the moral nature of the situation, rather than just being primers of the task ahead. We note that a previous study showed that a simple nudge at the end of a Dictator Game instruction (i.e., “Note that he relies on you”) increased Dictator’s donations (Brañas-Garza, 2007); however, these experiments were conducted in a classroom or in a lab, not online, and the means of delivery may have affected the level of engagement of the participants.

The present study provides some interesting findings, but it has limitations. First, the scarcity scales were developed *ad hoc* for the study and were not validated on a separate sample. Given that the goal was to capture conditions that were heavily influenced by the fast-changing socio-political situation, there were time constraints that prevented the development of a fully validated scale. Second, the number of measures obtained was relatively limited: for example, we limited the scarcity scales to 5 items and no measure of affect or risk-taking was included. The choice to limit the number of items administered was driven by the need to avoid burdening participants to improve the quality of responses. Third, the nature of the study was correlational rather than experimental, aiming to capture the perception of resource scarcity associated with the pandemic; at this point, no claim of causal relationship between scarcity and cooperation could be made. Forth, given the short amount of time we had to design and implement the study, and the aim to keep the situations described in the PGG tasks as relevant as possible to the ongoing lockdown situation, we focused more on real-life relevance and less on other aspects of the PGG, such as, for example, group size, which has been shown to play a key role on participants’ willingness to cooperate (Pereda et al., 2019). Likewise, since our approach was psychological and we aimed to prioritise relevance to the situation and ease of understanding, the structure of the games, and in particular of the PGG-time, slightly deviated from the standard PGG described in the economic literature, making it more challenging to compare these results to the ones in the literature. Although at the time of writing (May 2022) many Western Countries, also thanks to successful mass vaccination programs, are not experiencing strict lockdown restrictions anymore, investigations into pandemic-related scarcity will nevertheless still be relevant in the months, and years, to come. For this reason, future studies may want to investigate whether a perceived scarcity of socio-psychological wellbeing, elicited for example by framing messages in terms of lack of social connections caused by the pandemic, may boost cooperation (in the same fashion as Jordan et al., 2021 and Everett et al., 2020); on the other hand, framing messages in terms of lack of freedom of movement and time to elicit freedom scarcity may have the opposite effect. Moreover, future investigation into perceived scarcity may look at cooperation and competition for resources at the same time (e.g., Roux et al., 2015), to obtain a more detailed picture of prosocial behavior.

Overall, these findings provide some relevant theoretical insights into the relationship between scarcity and prosocial behavior. Future studies will determine whether framing messages by manipulating the perception of scarcity for different kinds of resource domains may have different effects on prosocial behavior, which will be relevant to inform public messaging.

## Data availability statement

The datasets presented in this study can be found in online repositories. The names of the repository/repositories and accession number(s) can be found in the article/Supplementary material.

## Ethics statement

The studies involving human participants were reviewed and approved by the Ethics Panel of the School of Applied Sciences at London South Bank University, with protocol number ETH1920-0152. The patients/participants provided their written informed consent to participate in this study.

## Author contributions

CC contributed to the design, the analysis, and took the lead in writing the manuscript. MC, EC, and ER contributed to the design, the analysis, and the writing of the manuscript. IH contributed to the design and the analysis. All authors contributed to the article and approved the submitted version.

## Funding

The study was funded by the School of Applied Sciences (data collection) and by the Library and Learning Resources (open access publication fees) of London South Bank University.

## Conflict of interest

The authors declare that the research was conducted in the absence of any commercial or financial relationships that could be construed as a potential conflict of interest.

## Publisher’s note

All claims expressed in this article are solely those of the authors and do not necessarily represent those of their affiliated organizations, or those of the publisher, the editors and the reviewers. Any product that may be evaluated in this article, or claim that may be made by its manufacturer, is not guaranteed or endorsed by the publisher.

## Footnotes

^1^ https://osf.io/n9wgp/?view_only=41f95e3fdd28449695d523db919f7a53

^2^ https://osf.io/k3h4n/?view_only=b26c9ad20ebb4f9ba7bbe1dcd09554e1
