# Supplementary material for: How perceived scarcity predicted cooperation during early pandemic lockdown
Source: Front Psychol. 2022 Oct 10;13:951757. doi: 10.3389/fpsyg.2022.951757 (PMC9589443; doi:10.3389/fpsyg.2022.951757)
Supplement: Supplementary file 1 [file Presentation_1.pdf]

Supplementary Materials

Considering that the median contribution is very high both for the PGG-money (£70) and for the PGG-time (9), we ran an additional logistic regression model on both outcome variables. We consider the median as the cut-off, and more specifically we divided responses into lower than or equal to the median value (<80 for PGG-money and <10 for PGG-time), and higher than the median value. Here we report the visual representation of the probabilities for each predictor, for money (Figure 1) and time (Figure 2). Results confirm those of the linear models:

- for the PGG-money, COS and scarcity of socio-psychological wellbeing significantly predict the likelihood of contributing all the money, with 52% and 55% of probability, respectively; MacArthur also positively predicted the contribution (52%); conversely, Sample negatively and significantly predicted the likelihood to contribute all the endowment (35%).
- For the PGG-time, scarcity of socio-psychological wellbeing positively and significantly predicted the likelihood of sacrificing all the allocated days (54%), whilst the scarcity of freedom negatively and significantly predicted the contribution (43%)

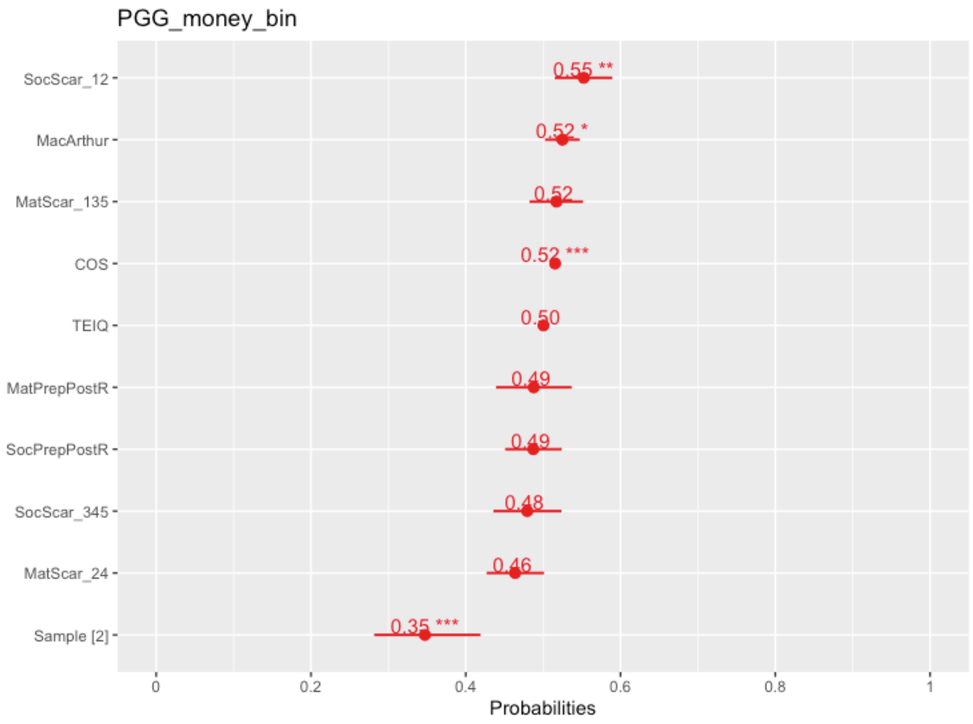

Figure 1. Probabilities of the effects of the predictors of the model (SocScar12 = Socio-psychological scarcity) on the likelihood to more than the median sum of money (70)

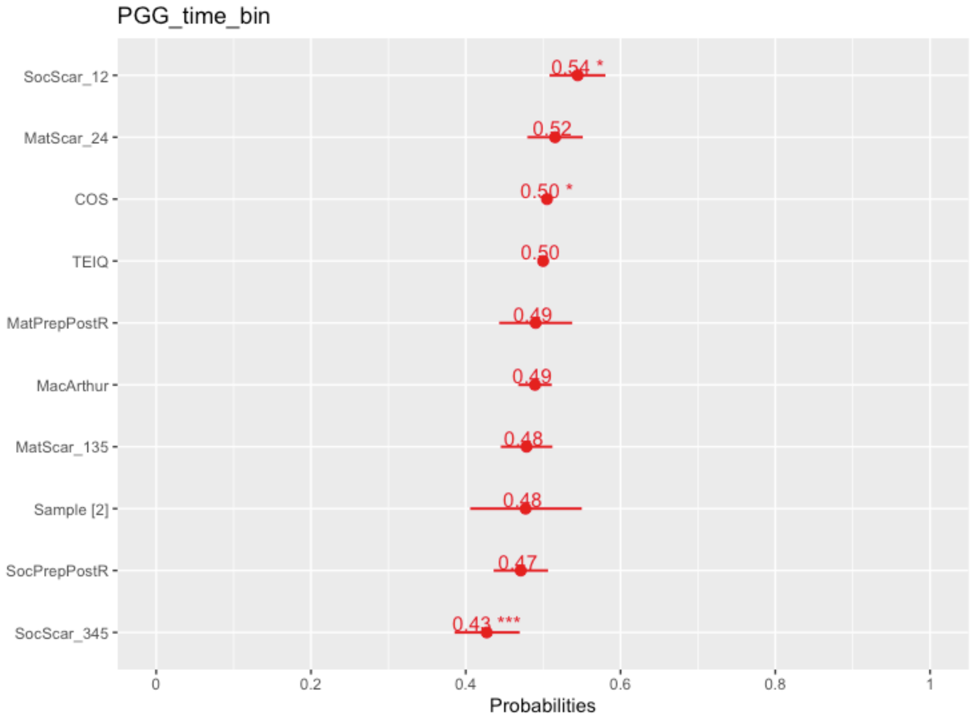

Figure 2. Probabilities of the effects of the predictors of the model (SocScar12: Scarcity of socio-psychological wellbeing; SocScar345: Scarcity of Freedom) on the likelihood to contribute more than the median allocated time (9)

We also ran two additional logit models, one for PGG-money and one for PGG_time, considering only demographic predictors to explore whether there were some factors that could predict the likelihood of contributing more than the median value. The only factor that positively and significantly predicted the amount contributed, both for the PGG-money (Figure 3) and for the PGG-time (Figure 4), was age.

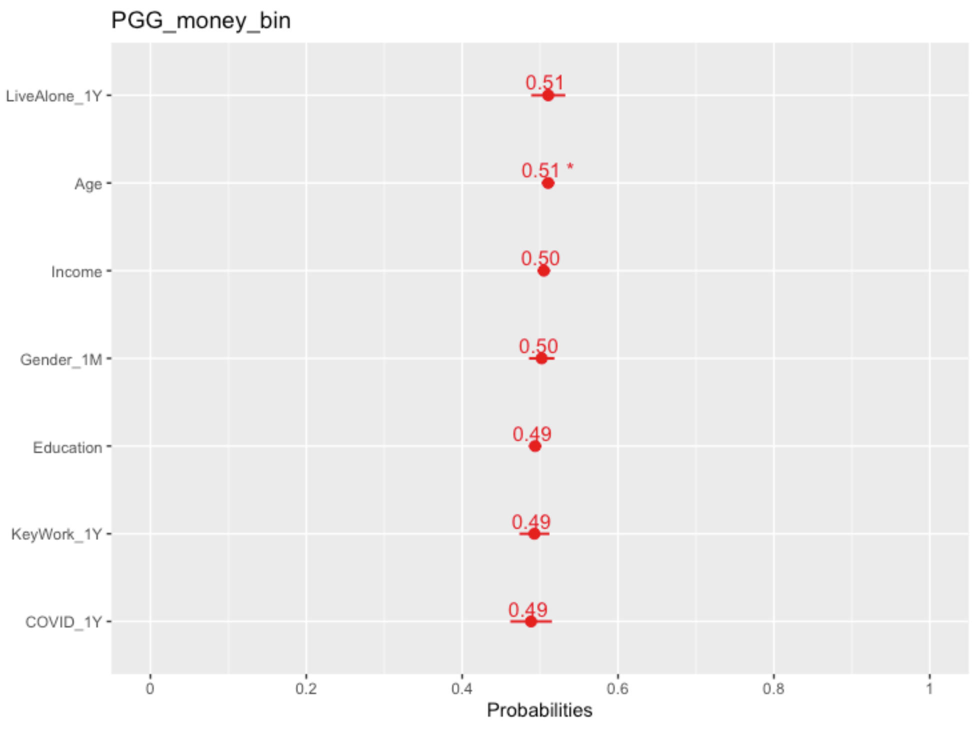

Figure 2. Probabilities of the effects of the predictors of the model (demographics) on the likelihood to contribute more than the median sum of money (70)

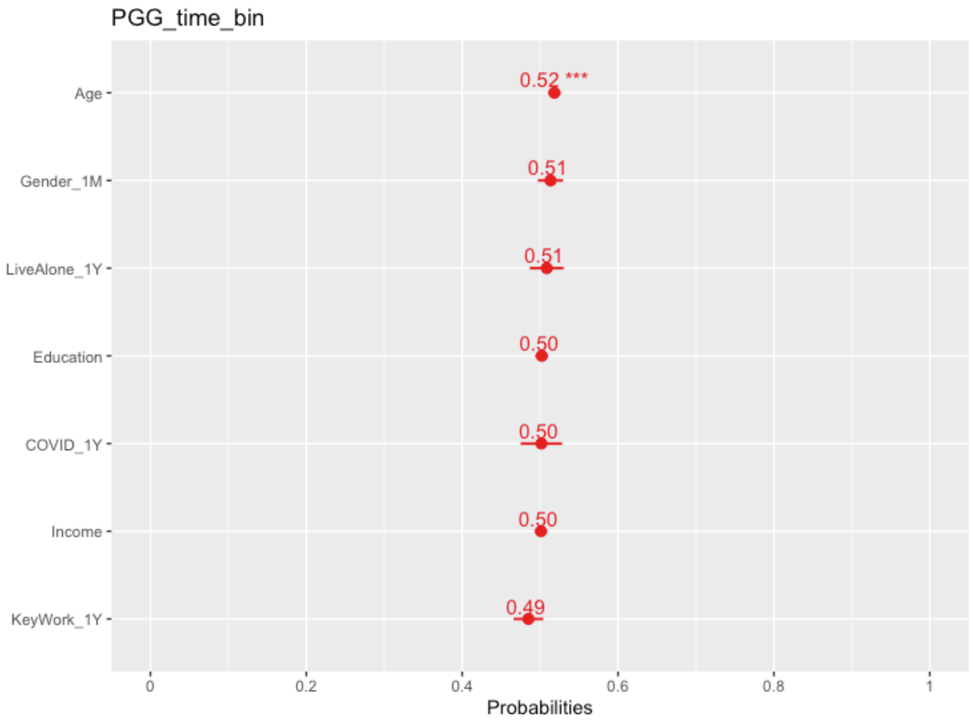

Figure 2. Probabilities of the effects of the predictors of the model (demographics) on the likelihood to contribute more than the median allocated time (9)
